# Predicting social anxiety in young adults with machine learning of resting-state brain functional radiomic features

**DOI:** 10.1038/s41598-022-17769-w

**Published:** 2022-08-17

**Authors:** Byung-Hoon Kim, Min-Kyeong Kim, Hye-Jeong Jo, Jae-Jin Kim

**Affiliations:** 1 Department of Psychiatry, Yonsei University College of Medicine, Seoul, Republic of Korea; 2 Institute of Behavioral Sciences in Medicine, Yonsei University College of Medicine, Seoul, Republic of Korea

**Keywords:** Neuroscience, Computational neuroscience

## Abstract

Social anxiety is a symptom widely prevalent among young adults, and when present in excess, can lead to maladaptive patterns of social behavior. Recent approaches that incorporate brain functional radiomic features and machine learning have shown potential for predicting certain phenotypes or disorders from functional magnetic resonance images. In this study, we aimed to predict the level of social anxiety in young adult participants by training machine learning models with resting-state brain functional radiomic features including the regional homogeneity, fractional amplitude of low-frequency fluctuation, fractional resting-state physiological fluctuation amplitude, and degree centrality. Among the machine learning models, the XGBoost model achieved the best performance with balanced accuracy of 77.7% and F1 score of 0.815. Analysis of input feature importance demonstrated that the orbitofrontal cortex and the degree centrality were most relevant to predicting the level of social anxiety among the input brain regions and the input type of radiomic features, respectively. These results suggest potential validity for predicting social anxiety with machine learning of the resting-state brain functional radiomic features and provide further understanding of the neural basis of the symptom.

## Introduction

Social anxiety is defined as fear or anxiety about social situations in which there is a possible scrutiny by others^1^. This type of anxiety is thought of as a helpful reaction to encourage one’s appropriate social behavior when it is within an adaptive range^2^. However, its excess can lead to functional disability, such as avoiding events that involve social interactions, and thus people with marked social anxiety are diagnosed with social anxiety disorder (SAD)^3^. The prevalence of SAD in young adults is reported to be as high as 18%, which is the most diagnosed among anxiety disorders^4^. Considering that there are subjects with subclinical symptoms of social anxiety which can cause discomfort and disturbance in daily life, understanding social anxiety in the general population is an important topic for the promotion of mental health^1^.

The biological basis of anxiety is thought to be underpinned within the limbic system^5^. In particular, the amygdala controls the provocation of anxiety and innate fear responses^6^, and closely communicates with the hippocampus for constituting the function of emotional responses to an engraved fear stimulus^7^. These two limbic structures are specifically involved in provoking anxiety from the low-level, meaning that the provoked emotion and its responses are instinctive rather than cognitive^8^. For the formation of social anxiety, brain regions that take part in higher-level functions are also known to be involved along with the limbic system^9^. For example, the orbitofrontal cortex (OFC) is a cortical region that functions in reward value evaluation, decision-making, and emotion processing during social situations^10,11^. In particular, impairment of reward value evaluation related to social situations can undermine and exaggerate the expected negative outcome from failure in social interaction^12^. The understanding of exaggerated social anxiety arising from functional impairment of the OFC is further supported by neuroimaging evidence that this cortical region exhibits interplay with the low-level limbic regions, including the amygdala^13–15^.

Resting-state functional magnetic resonance imaging (fMRI) has been used to explore network-level relevance to social anxiety across brain regions^16,17^. The default mode network (DMN) is a key network that is active during resting-state and is grounded on the posterior cingulate cortex and precuneus^18^. This network takes part in self-referential processing and rumination, and hyperactivity of these functions are suggested to be correlated with the level of negative cognition towards oneself^19^. This negative cognition can also affect the sensitivity to negative evaluation of others, leading to increased social anxiety^20^. Unsurprisingly, many resting-state fMRI studies have confirmed the involvement of the DMN in the pathophysiology of social anxiety and SAD^21,22^.

Investigating the neural correlates using neuroimaging modalities has provided researchers with opportunities to identify social anxiety-related brain regions through statistical approaches. However, few attempts have been made to infer (i.e. classify or regress) the level of social anxiety by training a model based on one’s neural characteristics. One previous study employed multivoxel pattern analysis (MVPA)^23^ and support vector machines (SVMs)^24^ for classifying patients with SAD from healthy controls in a total of 26 participants, which is a small sample size to train a machine learning model^25^. There were other studies that tried to classify SAD with machine learning models, but these studies did not take any functional measurement of the brain into account for the classification task^26,27^.

Radiomics is a recent field of research that focuses on extracting information containing specific patterns related to certain phenotypes from medical image data^28,29^. Approaches to incorporating information extracted from fMRI scan images, thought of as functional radiomic features, with machine learning models have shown promising results in the classification of psychiatric or neurological disorders^30,31^. We denote the term resting-state brain functional radiomic features as the set of information extracted from resting-state functional neuroimage data, which include the regional homogeneity (ReHo), fractional amplitude of low-frequency fluctuation (fALFF), fractional resting-state physiological fluctuation amplitude (fRSFA), and degree centrality (DC). The ReHo is a measure of brain activity that reflects the local consistency of blood oxygen level dependence (BOLD) through time^32^. The fALFF and fRSFA are resting-state measures that represent spontaneous fluctuations arising from neuronal activity and the level of hemodynamic responses, respectively^33,34^. The DC incorporates the number or strength of connection that is incident at each voxel of the brain^35^. Extracting these functional radiomic features from the BOLD signal incorporates previous knowledge regarding the physiology of the brain, such as low-frequency oscillations, adding plausibility and power when used as an input feature for training a machine learning model. Although these measures have been separately studied to uncover a neural basis of various phenotypic characteristics and disorders^16,36^, there has not been an approach which tried to train a model that considers these measures at the same time.

In the current study, we hypothesized that the resting-state brain functional radiomic features within the brain regions related to social anxiety would hold relevant neurological information that can be used to determine the level of social anxiety. This is based on a large body of previous evidence that alterations in the functional radiomic features such as the ReHo^36,37^, fALFF^38,39^, and DC^40,41^ are accompanied by a change in the level of social anxiety. Accordingly, the aim of this study was to evaluate the potential validity of machine learning models with over 100 young adult participants for classifying them into either low or high social anxiety group with machine learning models based on the resting-state brain functional radiomic features extracted from the fMRI data. In addition, we estimated the relative importance of the input radiomic features for determining the final prediction, deriving further neuroscientific understanding about social anxiety in these young adults.

## Results

### Participant demographics and psychometric Scale scores

**Figure 1:**
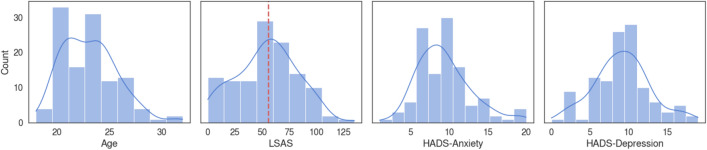
Histogram plot of age and psychometric scale scores of the participants. Red dotted vertical line on the LSAS plot indicates the median score (median 55.5), which served as the cutoff for separating the two groups. Abbreviations: LSAS, Liebowitz Social Anxiety Scale; HADS-A, anxiety score of the Hospital Anxiety and Depression Scale; HADS-D, depression score of the Hospital Anxiety and Depression Scale.

Average and standard deviation of age, Liebowitz Social Anxiety Scale (LSAS), Hospital Anxiety and Depression Scale related to anxiety (HADS-A) and depression (HADS-D) scores of the 116 participants and each group of low social anxiety participants (LSA) and high social anxiety participants (HSA) are presented in Table 1. Median of the LSAS score was 55.5, which served as the cutoff for separating the LSA and HSA groups. Age (t_114_ = − 1.34, p = 0.182) and level of depression measured by the HADS-D score (t_114_ = 0.91, p = 0.366) did not differ between the two groups on the independent t-test, whereas level of anxiety measured by the HADS-A score (t_114_ = 3.60, p < 0.001) differed between the two groups significantly. Histogram plot of the age and psychometric scale scores of the 116 participants are provided in Fig. 1. Since the HADS-A score was significantly different between the two groups, we further performed Pearson’s correlation analysis between the HADS-A score and the LSAS score and revealed a statistically significant correlation (r = 0.32, p = 0.001). However, within-group correlation in the LSA group (r < 0.01, p = 0.985) and the HAS group (r = 0.21, p = 0.104) were not statistically significant. The difference in the correlation coefficient between the two groups compared by the z-test of the Fisher’s r-to-z transformed coefficient values was not significant either (z = 1.13, p = 0.256), showing that the LSAS score and the HADS-A scores represent distinct type of anxiety in the group-level analysis.

**Table 1:** Participant demographics and psychometric scale results.

	All (n = 116)	LSA (n = 58)	HSA (n = 58)	t (χ^2^)	p-value
Age	23.2 ± 2.6	23.5 ± 2.7	22.8 ± 2.5	− 1.34	0.182
Sex (F/M)	79/37	33/25	46/12	5.79	0.016
LSAS	54.3 ± 28.3	31.7 ± 17.4	76.9 ± 16.5	14.36	< 0.001
HADS-A	9.4 ± 3.5	8.3 ± 3.3	10.5 ± 3.4	3.60	< 0.001
HADS-D	9.2 ± 3.7	8.9 ± 3.8	9.5 ± 3.6	0.91	0.366

*LSA* low social anxiety group, *HAS* high social anxiety group, *F* female, *M* male, *LSAS* Liebowitz Social Anxiety Scale, *HADS-A* anxiety score of the Hospital Anxiety and Depression Scale, *HADS-D* depression score of the Hospital Anxiety and Depression Scale.

### Machine learning experiment result

**Figure 2:**
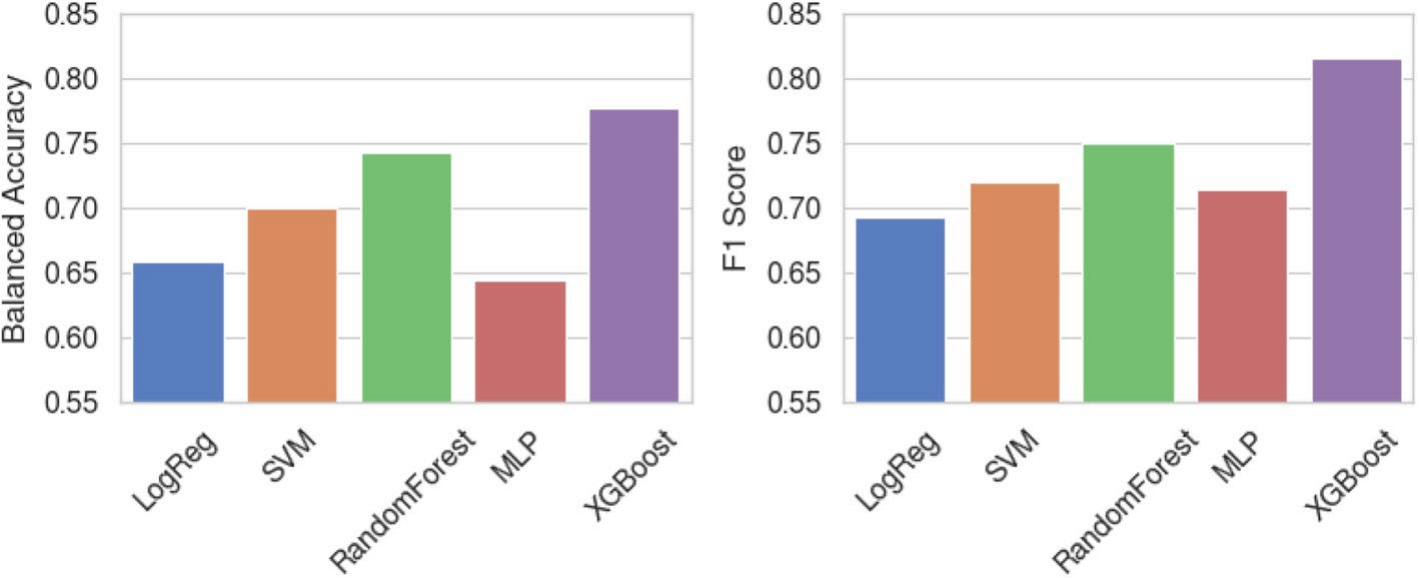
Bar plot of classification performance of the machine learning models.

As presented in Table 2 and Fig. 2, the accuracy of the logistic regression (LogReg), SVM, random forest (RF), multi-layer perceptron (MLP), and the extreme gradient boosting (XGBoost) for classifying the social anxiety group was 0.652, 0.696, 0.739, 0.652, and 0.783, respectively. The balanced accuracy, which is a metric that compensates the class imbalance problem, of LogReg, SVM, RF, MLP, and XGBoost resulted in 0.659, 0.701, 0.742, 0.644, and 0.777, respectively. The F1 score, which reflects both the sensitivity and specificity of the model by computing the harmonic mean of the precision and recall scores, also resulted in a similar trend with the accuracy and the balanced accuracy, demonstrating 0.692, 0.720, 0.750, 0.714 and 0.815 for the LogReg, SVM, RF, MLP, and XGBoost models, respectively. It could be seen that the XGBoost model resulted in the best classification performance on the test data in terms of all three metrics evaluated. The average prediction probability suggests that the average uncertainty of the model prediction is the lowest for the LogReg, and the highest for the XGBoost. Standard deviation of the model prediction uncertainty was the lowest for the XGBoost model. The final training hyperparameters of the best XGBoost model selected with the exhaustive grid search were (1) booster: gblinear, (2) alpha: 0.1, and (3) lambda: 1.0, suggesting that the gradient boosting of linear model ensemble may outperform the gradient boosting of tree-based model ensemble. The Dummy model represents the chance-level baseline classifier where no estimator is actually trained.

**Table 2:** Classification performance of the machine learning models.

Model	Accuracy	Balanced accuracy	F1 score	Average prediction probability
Dummy	0.478	0.5	0.0	0.505 ± 0.000
LogReg	0.652	0.659	0.692	0.902 ± 0.123
SVM	0.696	0.701	0.720	0.645 ± 0.043
RF	0.739	0.742	0.750	0.702 ± 0.129
MLP	0.652	0.644	0.714	0.858 ± 0.150
XGBoost	0.783	0.777	0.815	0.642 ± 0.051

*LogReg* logistic regression, *SVM* support vector machine, *MLP* multi-layer perceptron, *RF* random forest, *XGBoost* extreme gradient boosting.

**Figure 3:**
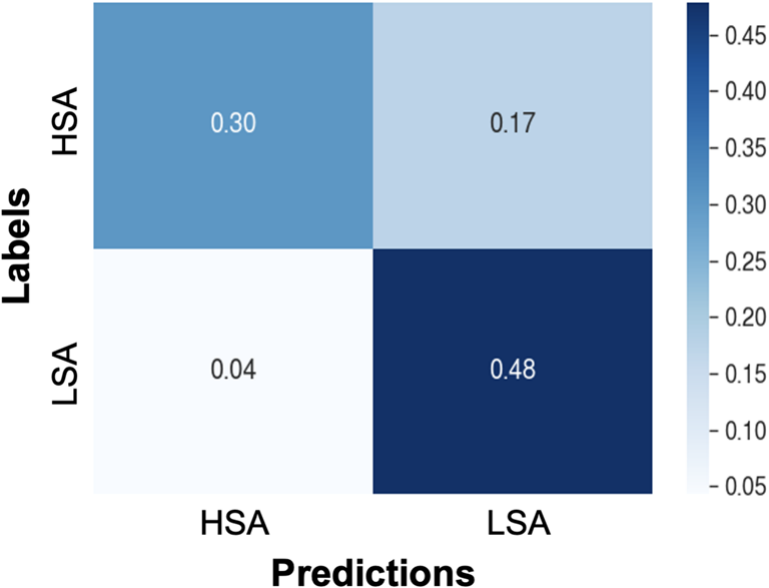
Confusion matrix of the extreme gradient boosting (XGBoost) model prediction. Annotations of each box represents the ratio of samples in that category.

Predictions of the XGBoost model with respect to the correct labels are plotted as a confusion matrix in the Fig. 3 to help understand the errors in the prediction. It can be seen that the HSA samples which are classified as the LSA (17%) constitute about 4 times more than the opposite error case (4%).

### Resting-state radiomic feature importance

**Figure 4:**
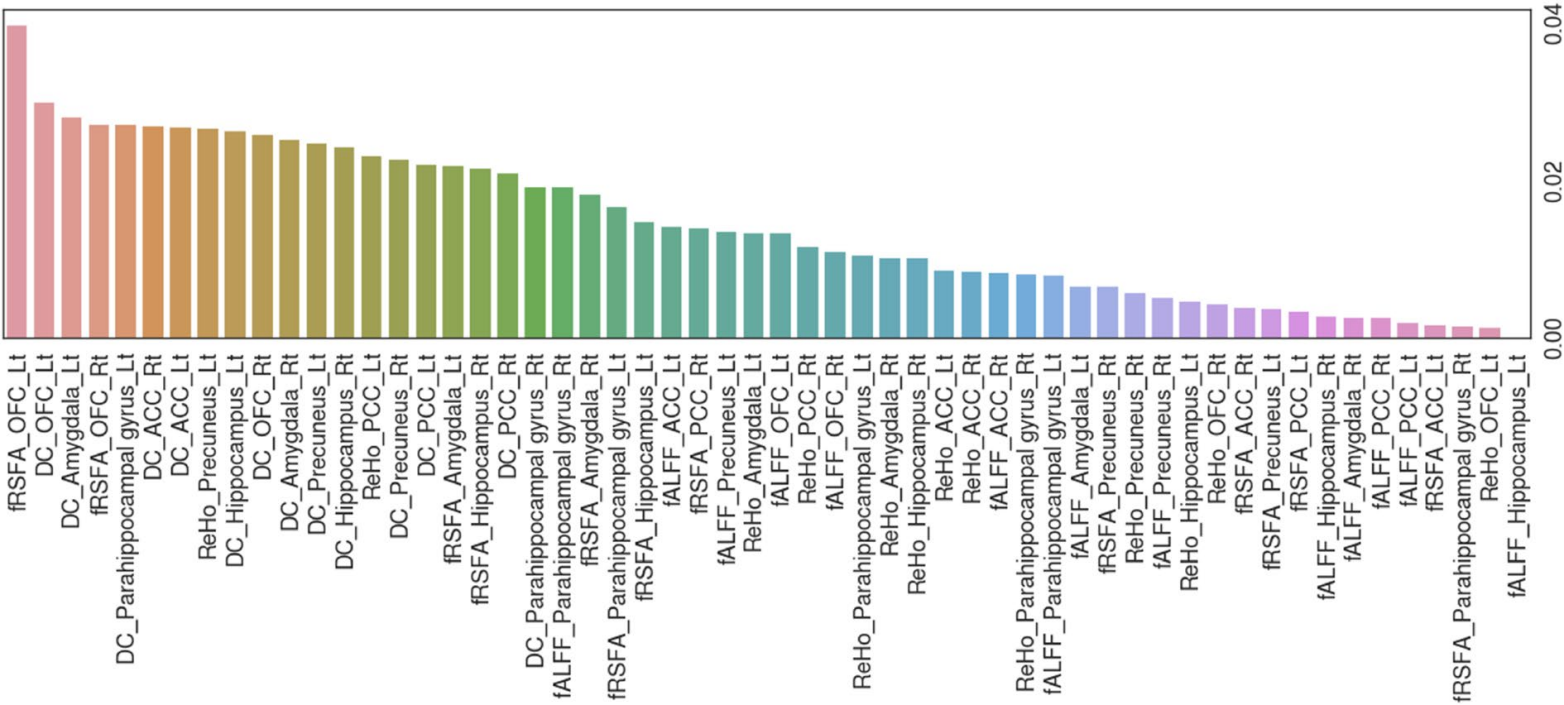
Feature importance bar plot all input resting-state brain functional radiomic features. The importance value is estimated by SHapley Additive exPlanation (SHAP) feature importance method on the eXtreme Gradient Boosting (XGBoost) model with the test data split. *Rt* right, *Lt* left, *DC* degree centrality, *fRSFA* fractional resting-state physiological fluctuation amplitude, *fALFF* fractional amplitude of low-frequency fluctuation, *ReHo* regional homogeneity, *PCC* posterior cingulate cortex, *OFC* orbitofrontal cortex, *ACC* anterior cingulate cortex.

**Figure 5:**
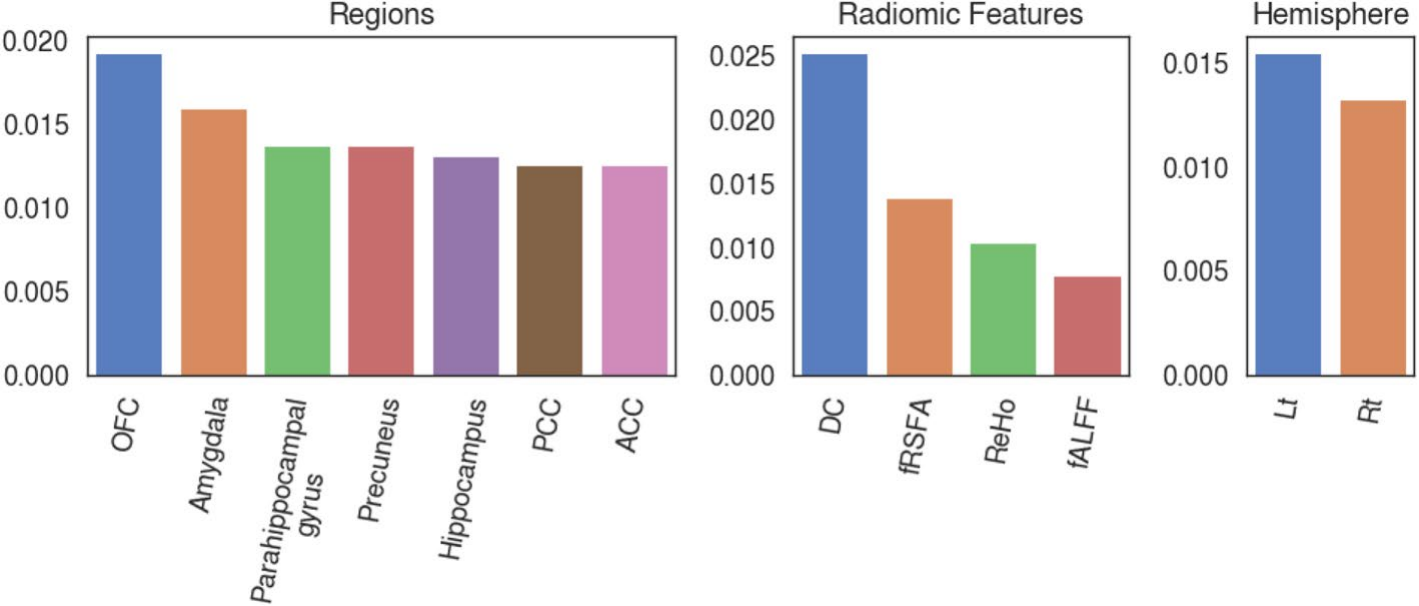
Sum of absolute SHapley Additive exPlanation (SHAP) feature importance for each region, radiomic feature, and the two hemispheres. *OFC* orbitofrontal cortex, *PCC* posterior cingulate cortex, *ACC* anterior cingulate cortex, *DC* degree centrality, *fRSFA* fractional resting-state physiological fluctuation amplitude, *ReHo* regional homogeneity, *fALFF* fractional amplitude of low-frequency fluctuation, *Rt* right, *Lt* left.

The relative importance of input resting-state brain functional radiomic features are provided as a barplot in Fig. 4. Among the 56 features, the fRSFA of the left OFC, the DC of the left OFC, and the DC of the left amygdala were the three most relevant features to the social anxiety classification task. Contribution of each region, radiomic feature, or hemisphere to the importance values are plotted in Fig. 5. In terms of the importance across different regions, the OFC resulted in the highest importance value sum, followed by the Amygdala, parahippocampal gyrus, precuneus, hippocampus, posterior cingulate cortex (PCC), and anterior cingulate cortex (ACC). Among the radiomic features, the DC showed the highest importance value sum, followed by the fRSFA, ReHo, and fALFF. Lastly, the left hemisphere demonstrated higher importance value sum than the right hemisphere.

## Discussion

This study aimed to classify young adults into either the low or high social anxiety group with machine learning models based on the resting-state brain functional radiomic features extracted from the fMRI scan images. The functional radiomic features included the ReHo, fALFF, fRSFA, and DC of the resting-state fMRI within the OFC, ACC, PCC, hippocampus, parahippocampal gyrus, and amygdala. Of the six machine learning models tested, the XGBoost model demonstrated the best performance with balanced accuracy of 0.777 and F1 score of 0.815, outperforming LogReg, SVM, RF, and MLP estimators. From the feature importance analysis, the DC was the most important radiomic feature and the OFC, amygdala, and parahippocampal gyrus were the top three important regions for the classification of social anxiety.

From the demographics and psychosocial scales, age of the participants enrolled in this study were 23.2 in average with standard deviation of 2.6. This distribution of age represents that the recruited participants only include young adults, and suggests that the results should not be interpreted as those of the general population. Nevertheless, considering that social anxiety is more prevalent in young adults and that aging itself can affect functional activity of the resting brain, focusing on social anxiety only in young adults can be a strength rather than a limitation. The distribution of sex between the LSA and the HSA groups were found to have the statistically significant difference. This finding can reflect the fact that the prevalence of SAD is greater in women than men, leading to a larger ratio of female participants who have high level of social anxiety^42,43^. We did not select the samples to stratify the gender ratio by evaluating the participant demographics, because it can introduce unwanted selection bias to our dataset. In other words, if the models are trained and evaluated from an in-house modified sample distribution, it can lead to a lower generalization capability in real-world applications. Even so, it should be noted that gender differences may have influenced the feature importance result, especially within the PCC and precuneus, regions of the DMN where such differences have been suggested^44^. Also, there may exist potential bias given that demographic variables such as gender or age are not included as the input feature.

The LSAS score was 54.3 in average with standard deviation of 28.3, suggesting that the recruited participants consisted of people with a wide range of social anxiety levels. A notable result from the psychometric scale analysis is that the two groups showed significant difference in not only the LSAS score, but also the HADS-A score. This reflects the trend that the participants with higher level of social anxiety also exhibit higher level of general state anxiety, and vice versa. This trend is confirmed by further correlation analysis which showed the significant correlation between the LSAS score and the HADS-A score in the participants. While it may be very difficult, or even impossible to delineate the correlation between social anxiety and general state anxiety, caution should be made in interpreting our results given that general state anxiety might have been a confounding factor.

Another notable point in the psychometric scale analysis is that there were participants with unexpectedly high level of social anxiety, who were not being evaluated or treated by a clinician. This existence of highly socially anxious people without a history of treatment may reflect the socio-cultural environment that makes them reluctant to receive psychiatric treatment. In particular, East Asian culture itself is known to affect people’s level of social anxiety^45,46^, and people with psychiatric difficulties tend to avoid seeking clinical help because of the stigma of mental illness until they become severe^47^. Given these situational factors, the prediction model of social anxiety can be effectively used as an unbiased screening tool without concerns regarding the stigmatization for having mental illness.

The classification experiments demonstrate that the XGBoost model achieved the best performance when compared to the other classifiers. The SVM model resulted in balanced accuracy of 0.701, which is slightly lower than, but comparable to, the previous work reporting balanced accuracy of 0.726 by incorporating the task-based fMRI features within the amygdala, ACC, hippocampus, insula, and parietal lobe trained with the SVM^25^. This discrepancy may come from the difference in the (1) type of extracted functional radiomic features, (2) selected brain regions for training the model, (3) presence of specific behavioral task during the fMRI scan, (4) hyperparameter settings for training the model, or (5) number of samples (n = 116 vs. n = 26) included in the study. Balanced accuracy of the XGBoost model showed 0.777, outperforming the LogReg, SVM, RF, and MLP classifiers trained in our experiments and also the result from the previous study^25^. The XGBoost classifier is an ensemble of multiple estimators optimized with the gradient boosting technique. The ensemble technique of multiple estimators along with the regularized objective function, shrinkage and subsampling helps prevent overfitting of the final model, which could partly explain the good performance of the XGBoost model over other models^48^. Even for the best XGBoost model, the performance for predicting social anxiety still has room to improve.

Importance of each input functional radiomic feature was computed and analyzed with the SHAP feature importance method in this study. Among the brain regions, the left OFC was found to be more important than others from the mean of absolute SHAP values (Fig. 4). Considering that the fRSFA reflects resting-state vascular reactivity of the region, salient resting-state activity feature within the OFC can be possibly related to the level of social anxiety, which the trained XGBoost model largely accounts for when performing the classification task. Importance of the left OFC in classifying the level of social anxiety is further emphasized by the fact that the second most important functional radiomic feature is also within the left OFC. Results from our experiments suggest that the DC of the left OFC and amygdala are two of the highly important regions for the social anxiety prediction (Fig. 4). The DC is a network metric that represents how many connections are present within the node of interest, related to the strength of functional connectivity^35^. These findings are concurrent with previous neuroimage evidence given that the OFC is one of the key regions in the arousal of social anxiety, which plays a central role as the mediator between the low-level limbic regions, such as the amygdala, and the higher-level network hubs, such as the PCC and precuneus. Aberrant resting-state function of the OFC in terms of reduced activity^49^, and abnormal functional/effective connectivity to the amygdala^14,15^ have been consistently reported in previous neuroimaging studies of SAD patients. The role of the OFC in social anxiety is related to the fact that the region takes part in high-level functioning of reward value evaluation in social situations^11^. To sum up, it can be interpreted that the XGBoost model appropriately takes the level of activity and connectivity within the OFC and amygdala into account, which is known to be related to the level of social anxiety in patients with SAD, for the social anxiety classification.

Some issues of the current study should be mentioned. Although our study included collection of fMRI data samples from over 100 young adult participants, by far the largest number among the machine learning studies of resting-state fMRI related to social anxiety, it should still be noted that a larger number of samples may provide a more convincing result in the future. Other limitations of this study can include that the demographic factor, such as age or gender, is not taken into account during training, which can affect the resting-state brain functional radiomic features. In addition, the neuroimaging data was acquired from two different sites. Although we have gone through an extensive pre-processing pipeline to minimize the multi-site effect, the possibility of multi-site effect may still be a limitation of this study.

Training machine learning models for the classification of one’s psychological phenotype has potential clinical benefits. Although the accuracy for prediction may be a little below the certain point that the method can actually be extended to clinical practice, the experimental results still hold the significance in that they validate the potential possibility of radiomics-based machine learning for screening young adults with high social anxiety who were not aware of, or not able to seek clinical help. However, it should also be noted that there also exists potential negative societal impact related to privacy concerns that arise from abuse or misuse of decoding methods. Although the accuracy and capability of our method are yet behind the level that can be abused or misused, these societal concerns should still be considered.

In conclusion, we trained machine learning models with the resting-state brain functional radiomic features from young adult participants to predict the level of social anxiety, and confirmed validity of the approach by experiments. The feature importance analysis results demonstrated that the brain regions responsible for high-level functions were more important to the prediction of social anxiety than those related to low-level limbic functions. Our approach based on machine learning with the functional radiomic features for predicting the level of social anxiety may potentially be a useful screening tool for people with high level of social anxiety, and possibly other psychopathologic phenotypes in the future.

## Methods

### Participants

Young adult participants were recruited from advertisement via the Internet, and were evaluated by a trained psychiatrist whether they met the exclusion criteria of (1) any history of diagnosis or treatment of major psychiatric disorder, (2) inability to undergo the MRI scanning, (3) significant history of medical condition or neurological disorder, (4) pregnancy, and (5) left-handedness. It should be noted that only participants not receiving psychiatric treatment were included. There were 125 participants included at this step, of which 9 participants were dropped out from the final analysis due to willingness to withdraw from the study or severe artifacts in the acquired MRI data, leaving a total of 116 participants in the sample. Informed consent was obtained from all participants and the study was approved by the Institutional Review Board (IRB) of Yonsei University Gangnam Severance Hospital (3-2017-0046; date of approval: Mar 29, 2017). Whole process of this research was carried out in accordance with the Declaration of Helsinki.

### Psychometric evaluation

The LSAS^50^, which is a self-reporting four-point Likert scale with 24 items, was obtained from all participants to evaluate the level of social anxiety. Levels of general anxiety and depression are further measured by the self-reported HADS-A and HADS-D^51^. Participants were classified into the LSA group or HSA group according to whether their LSAS scores were lower or higher than the median value of all participants.

### Neuroimage data acquisition and preprocessing

The neuroimage data were collected from two different sites with magnetic resonance imaging facilities, based on the participant location. Images were acquired at the first site, using a 3.0 T MRI scanner (Ingenia CX, Philips, Best, the Netherlands) with a 32-channel head coil. For each participant, anatomical images were obtained in the coronal direction using a 3D T1-weighted fast gradient echo sequence (matrix size, 224 × 224; number of slices, 220; slice thickness, 1 mm; echo time, 4.6 ms; repetition time, 9.9 ms; and flip angle, 8°). The resting-state fMRI scans were acquired with the multiband SENSitivity Encoding (SENSE) sequence (matrix size, 96 × 93; field of view, 216 mm; number of slices, 60; slice order, bottom-up and interleaved; slice thickness, 2.4 mm; echo time, 30 ms; repetition time, 800 ms; flip angle, 52°; multiband factor, 6; and SENSE factor, 1). At the second site, the images were collected using Siemens Magnetom Verio 3 T scanner (Siemens Medical Solutions, Erlangen, Germany). Structural T1 weighted images were obtained with a 3D spoiled-gradient-recall sequence (matrix size, 256 × 256; number of slices, 176; slice thickness, 1 mm; echo time, 2.46 ms; repetition time, 1900 ms; and flip angle, 9°). The resting-state functional images were acquired with a gradient echo planar imaging sequence (matrix size, 64 × 64; number of slices, 30; slice thickness, 3 mm; echo time, 30 ms; repetition time, 2000 ms; and flip angle, 90°).

All data preprocessing and experiments were performed on a local Linux workstation equipped with Intel Core i7-8700 CPU @ 3.20 GHz × 12, 64 GB RAM, and NVIDIA GeForce RTX 2070. The T1-weighted and resting-state fMRI DICOM images were first converted into gzipped nifti files, and were renamed, organized to follow the BIDS standard specification. Preprocessing of these images which included skull-stripping, surface reconstruction, co-registration, motion correction, resampling, normalization, and segmentation were performed using fMRIPrep 20.2.3^52^, which is based on Nipype 1.6.1^53^. Further details on the neuroimage data preprocessing can be found on the Supplementary material.

### Resting-state brain functional radiomic feature extraction

**Figure 6:**
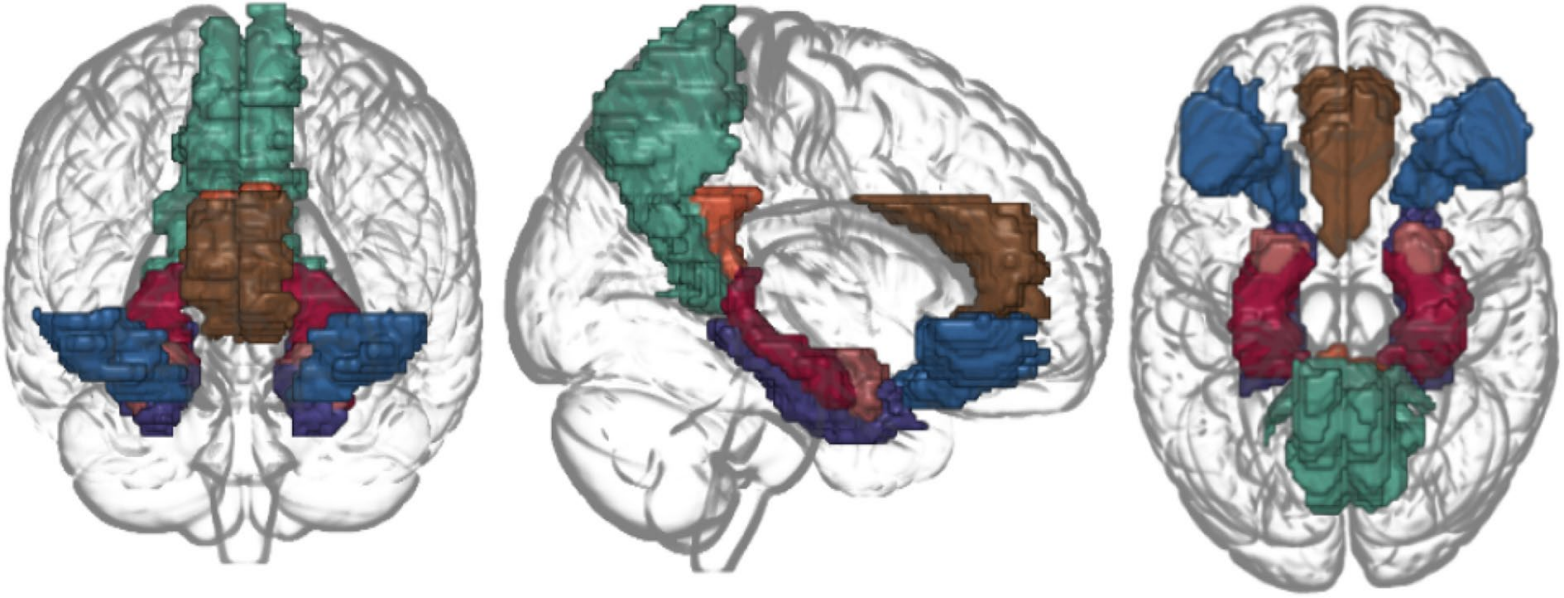
Social anxiety-related brain regions included in the current study. The regions were the orbitofrontal cortex (blue), anterior cingulate cortex (brown), posterior cingulate cortex (orange), precuneus (green), hippocampus (red), parahippocampal gyrus (purple), and amygdala (pink).

We focused on the resting-state brain functional radiomic features including the ReHo, fALFF, fRSFA, and DC. The radiomic features were extracted with the AFNI^54^ using functions 3dReHo, 3dRSFC, and 3dDegreeCentrality applied on the fully pre-processed 3d resting-state fMRI data across time. Further details on the radiomic feature definition and extraction are elaborated in the Supplementary material.. Radiomic feature value of regions of interest (ROIs) of the brain was computed by taking the average of the voxels within 14 selected regions from the automated anatomical labeling (AAL) atlas^55^ using the 3dROIstats function. The selected brain areas included bilateral regions of the OFC, ACC, PCC, precuneus, hippocampus, parahippocampal gyrus, and amygdala (Fig. 6). Thus, the final radiomic feature of a subject constituted a vector with 56 elements (7 regions × 4 radiomic features × 2 hemisphere sides).

### Dataset split and preprocessing

Dataset splitting and preprocessing pipeline was implemented with pandas (v1.2.3) and the scikit-learn (v0.24.1) library^56^ based on Python (v3.8.5). First, each sample was labeled with either ‘low’ or ‘high’ class based on the median LSAS score using the ‘qcut’ function of the pandas library. Second, the length 56 input feature vectors and the label pairs from 116 subjects were random-stratified split into the train and the test datasets using the ‘train_test_split’ function of scikit-learn, including 93 and 23 samples, respectively. To stabilize the training process, we fitted a ‘StandardScaler’ object of scikit-learn to the training input features, and transformed both the training and test input feature samples. This process calculates the mean and standard deviation of each feature from the train dataset, and apply shifting and scaling to the train and test datasets based on the calculated mean and standard deviation. The labels were encoded into an integer using the ‘LabelEncoder’ object of scikit-learn. The final number of samples in each split and group is provided in the Table 3.

**Table 3:** Number of samples in each group and split of the experiment dataset.

Split	LSA	HSA	Total
Train	46	47	93
Test	12	11	23
Total	58	58	116

*LSA* low social anxiety group, *HAS* high social anxiety group.

### Machine learning models and experiments

Machine learning models were implemented, trained, and tested with the scikit-learn (v0.24.1)^56^ library. We experimented six classification models including the Dummy, LogReg, SVM, RF, MLP, and eXtreme Gradient Boosting (XGBoost), which are machine learning methods widely used in academic research and industry. Here, the Dummy classifier was included to represent the baseline. Given that the model performance can be dependent on the hyperparameter settings, optimal hyperparameter of each model was selected using the `GridSearchCV` object on the training dataset. This object performs k-fold cross validation on the training dataset for every possible combination (grid) of the hyperparameters and allows to select the hyperparameter based on the average validation dataset performance score. We performed fivefold cross validation and the performance score was measured with the F1 score. The possible combinations (grid) of the training hyperparameters for each model are provided in the Supplementary material.. With the best six trained models (one per model type) selected from the ‘GridSearchCV’, accuracy, balanced accuracy, and F1 score were computed on the test dataset prediction to evaluate the final performance. To present the uncertainty of the model predictions, we also report the average and standard deviation of the positively predicted class probability.

### Feature importance estimation with shapley additive explanations

Importance of the input features was estimated using the SHapley Additive exPlantations (SHAP) feature importance method^57^ on the test dataset to investigate the relevance of each resting-state radiomic feature with the social anxiety classification task. The SHAP is a model-agnostic feature importance analysis method employing game-theoretic approach. Contribution of each input features to the final model prediction can be quantified with the SHAP value, and we estimate the feature importance as the average of absolute SHAP value computed across the test dataset samples^57^. To delineate the contribution of each region, radiomic feature, or hemisphere, we summed up the SHAP feature importance values from the same region, radiomic feature, or hemisphere and analyze the contribution of these features separately. We further evaluate the coefficient weights of the XGBoost model as the baseline feature importance estimate and show that the SHAP feature importance and the coefficient weights are highly correlated in the Supplementary material., which supports the robustness of the model explanation.

## Supplementary Information

Supplementary Information.

## Data Availability

Data requests should be directed to the corresponding author. Code is available at: https://github.com/egyptdj/fmri-radiomic-ml-sad/.
