# Supplementary material for: Predicting social anxiety in young adults with machine learning of resting-state brain functional radiomic features
Source: Sci Rep. 2022 Aug 17;12:13932. doi: 10.1038/s41598-022-17769-w (PMC9385624; doi:10.1038/s41598-022-17769-w)
Supplement: Supplementary file 1 — Supplementary Information. [file 41598_2022_17769_MOESM1_ESM.docx]

**Supplementary material: Predicting Social Anxiety in Young Adults with Machine Learning of Resting-State Brain Functional Radiomic Features**

**Details of the Neuroimage Preprocessing Step**

*Anatomical Data Preprocessing*

T1-weighted (T1w) image was corrected for intensity non-uniformity (INU) with N4BiasFieldCorrection (Tustison et al. 2010), distributed with ANTs 2.3.3 (Avants et al. 2008, RRID:SCR_004757), and used as T1w-reference throughout the workflow. The T1w-reference was then skull-stripped with a Nipype implementation of the antsBrainExtraction.sh workflow (from ANTs), using OASIS30ANTs as target template. Brain tissue segmentation of the cerebrospinal fluid (CSF), white-matter (WM) and gray-matter (GM) was performed on the brain-extracted T1w using FAST (FSL 5.0.9, RRID:SCR_002823, Zhang et al. 2001). Brain surfaces were reconstructed using RECON-ALL (FreeSurfer 6.0.1, RRID:SCR_001847, Dale et al. 1999), and the brain mask estimated previously was refined with a custom variation of the method to reconcile ANTs-derived and FreeSurfer-derived segmentations of the cortical gray-matter of Mindboggle (RRID:SCR_002438, Klein et al. 2017). Volume-based spatial normalization to one standard space (MNI152NLin2009cAsym) was performed through nonlinear registration with antsRegistration (ANTs 2.3.3), using brain-extracted versions of both T1w reference and the T1w template. The following template was selected for spatial normalization: ICBM 152 Nonlinear Asymmetrical template version 2009c (Fonov et al. 2009; RRID:SCR_008796; TemplateFlow ID: MNI152NLin2009cAsym).

*Functional Data Preprocessing*

A reference volume and its skull-stripped version were generated using a custom methodology of fMRIPrep. Susceptibility distortion correction (SDC) was omitted. The BOLD reference was then co-registered to the T1w reference using bbregister (FreeSurfer) which implements boundary-based registration (Greve and Fischl 2009). Co-registration was configured with six degrees of freedom. Head-motion parameters with respect to the BOLD reference (transformation matrices, and six corresponding rotation and translation parameters) were estimated before any spatiotemporal filtering using mcflirt (FSL 5.0.9, Jenkinson et al. 2002). The BOLD time-series (including slice-timing correction when applied) were resampled onto their original, native space by applying the transforms to correct for head-motion. These resampled BOLD time-series were referred to as preprocessed BOLD in original space, or just preprocessed BOLD. The BOLD time-series were resampled into standard space, generating a preprocessed BOLD run in MNI152NLin2009cAsym space. First, a reference volume and its skull-stripped version were generated using a custom methodology of fMRIPrep. Several confounding time-series were calculated based on the preprocessed BOLD: framewise displacement (FD), DVARS and three region-wise global signals. FD was computed using two formulations following Power (absolute sum of relative motions; Power et al. 2014) and Jenkinson (relative root mean square displacement between affines; Jenkinson et al. 2002). FD and DVARS were calculated for each functional run, both using their implementations in Nipype (following the definitions by Power et al. 2014). The three global signals were extracted within the CSF, WM, and whole-brain masks. Additionally, a set of physiological regressors were extracted to allow for component-based noise correction (CompCor; Behzadi et al. 2007). Principal components were estimated after high-pass filtering the preprocessed BOLD time-series (using a discrete cosine filter with 128s cut-off) for the two CompCor variants: temporal (tCompCor) and anatomical (aCompCor). tCompCor components were then calculated from the top 2% variable voxels within the brain mask. For aCompCor, three probabilistic masks (CSF, WM and combined CSF+WM) were generated in anatomical space. The implementation differed from that of Behzadi et al. in that instead of eroding the masks by 2 pixels on BOLD space, the aCompCor masks were subtracted a mask of pixels that likely contain a volume fraction of GM. This mask was obtained by dilating a GM mask extracted from the FreeSurfer’s aseg segmentation, and it ensured components were not extracted from voxels containing a minimal fraction of GM. Finally, these masks were resampled into BOLD space and binarized by thresholding at 0.99 (as in the original implementation). Components were also calculated separately within the WM and CSF masks. For each CompCor decomposition, the k components with the largest singular values were retained, such that the retained components’ time series were sufficient to explain 50 percent of variance across the nuisance mask (CSF, WM, combined, or temporal). The remaining components were dropped from consideration. The head-motion estimates calculated in the correction step were also placed within the corresponding confounds file. The confound time series derived from head motion estimates and global signals were expanded with the inclusion of temporal derivatives and quadratic terms for each (Satterthwaite et al. 2013). Frames that exceeded a threshold of 0.5 mm FD or 1.5 standardised DVARS were annotated as motion outliers. All resamplings could be performed with a single interpolation step by composing all the pertinent transformations (i.e. head-motion transform matrices, susceptibility distortion correction when available, and co-registrations to anatomical and output spaces). Gridded (volumetric) resamplings were performed using antsApplyTransforms (ANTs), configured with Lanczos interpolation to minimize the smoothing effects of other kernels (Lanczos 1964). Non-gridded (surface) resamplings were performed using mri_vol2surf (FreeSurfer).

**Details of the Functional Radiomic Features**

The functional radiomic features employed in this study are further described in detail in this section. All measures were computed with functions provided in AFNI, and the computed measures were aggregated with the 3dROIstats function.

*Regional Homogeneity (ReHo)*

The regional homogeneity (ReHo) is a measure that can be used to estimate how similar (homogeneous) the timeseries are between adjacent voxels (region). The homogeneity is computed by the Kendall’s coefficient concordance $\boldsymbol{W}$, defined as:

$\boldsymbol{W=}\frac{\boldsymbol{\Sigma}{\mathbf{(}\mathbf{R}_{\boldsymbol{i}}\mathbf{)}}^{\boldsymbol{2}}\boldsymbol{-n}{\boldsymbol{(}\bar{\boldsymbol{R}}\boldsymbol{)}}^{\boldsymbol{2}}}{\frac{\boldsymbol{1}}{\boldsymbol{12}}\boldsymbol{K}^{\boldsymbol{2}}\boldsymbol{(}\boldsymbol{n}^{\boldsymbol{3}}\boldsymbol{-n)}}$

where$\mathbf{R}_{\boldsymbol{i}}$ is the sum rank of the $\boldsymbol{i}$th timepoint; where $\bar{\boldsymbol{R}}\boldsymbol{=(}\left( \boldsymbol{n+1} \right)\boldsymbol{K)/2}$ is the mean of the $\mathbf{R}_{\boldsymbol{i}}$’s; $\boldsymbol{K}$ is the number of timeseries within a measured cluster; $\boldsymbol{n}$ is the number of ranks. Here, a cluster is a set of adjacent voxels with number of voxels $\boldsymbol{K\in\{7,9,27\}}$. The ReHo is known to detect unpredicted hemodynamic responses that other model-driven approaches for capturing local area homogeneity fail to (Zang et al., 2004).

*Fractional Amplitude of Low-Frequency Fluctuation (fALFF)*

The fractional amplitude of low-frequency fluctuation (fALFF) is an improved measure of the amplitude of low-frequency fluctuation (ALFF), which represents the level of physiologically meaningful low-frequency fluctuation of the brain (Zou et al., 2008). For computing the ALFF, temporal band-pass filter (0.01<<0.08 Hz) is first applied. The band-pass filtered timeseries of each voxel is Fourier transformed, and the power spectrum is obtained. The ALFF is defined as the averaged square root of the power spectrum across the voxels. The fALFF is computed as a fraction of ALFF at the target frequency range (0.01<<0.08 Hz) over the entire frequency range (0.0<<0.25 Hz). The fALFF is known to improve the ALFF measure by suppressing unwanted signals within the resting-state fMRI, such as the signals from cistern areas.

*Fractional Resting-State Physiological Fluctuation Amplitude (fRSFA)*

The resting-state physiological fluctuation amplitude (RSFA) is a measure extracted from resting-state fMRI data that carries information about vascular reactivity of the brain (Kannurpatti and Biswal, 2008). It is defined as the BOLD signal change computed as the standard deviation across time. This measure can be used to scale the BOLD timeseries to account for the vascular reactivity, or to predict task-related event with respect to the resting-state (Kannurpatti et al., 2012). The fractional resting-state physiological fluctuation amplitude (fRSFA) is a fraction of RSFA at the frequency range (0.01<<0.08 Hz) over the entire frequency range (0.0<<0.25 Hz).

*Degree Centrality (DC)*

The degree centrality (DC) is a graph-theoretic network metric which represents number of edges arising from a node. The DC was computed as a voxel-wise measure in this study, where the Pearson’s correlation over the BOLD timeseries between every pair of voxels within the brain mask were first computed. Then, a threshold (r < 0.6) is applied to exclude possible connections that have arisen by chance. A voxel’s correlation with all other voxels is summarized by summing the correlation coefficients.

**Hyperparameter Grid Setting**

*LogReg*

- 'solver': ['newton-cg', 'lbfgs', 'liblinear', 'saga'],
- 'penalty': ['l1', 'l2', 'elasticnet'],
- 'C': [10^-4^, 10^-3^, 10^-2^, 10^-1^, 1, 10, 10^2^, 10^3^],
- 'max_iter': [10000]

*SVM*

- 'kernel': ['linear', 'rbf', 'poly'],
- 'C': [10-3, 10-2, 10-1, 1, 10, 102],
- 'gamma': [10-3, 10-2, 10-1, 1, 10, 102, 103]

*RF*

- 'n_estimators': [10, 25, 100],
- 'max_features': ['auto', 3, 9, 15],
- 'max_depth': [None, 10, 20, 30, 40, 50, 60, 70, 80, 90, 100, 110],
- 'min_samples_split': [2, 5, 10],
- 'min_samples_leaf': [1, 2, 4],
- 'bootstrap': [True, False]

*MLP*

- 'hidden_layer_sizes': [10, 50, 100],
- 'solver': ['lbfgs', 'sgd', 'adam'],
- 'alpha': [0.01, 0.1, 1.0, 10.0],
- 'batch_size': [8, 16],
- 'learning_rate': ['constant', 'invscaling', 'adaptive'],
- 'max_iter': [1000],
- 'warm_start': [False, True]

*XGBoost*

- 'booster': ['gbtree', 'gblinear', 'dart'],
- 'learning_rate': [0.1, 0.01, 0.001, 0.0001],
- 'n_estimators': [10, 25, 100],
- 'sampling_method': ['uniform', 'gradient_based'],
- 'max_depth': [None, 10, 20, 30, 40, 50, 60, 70, 80, 90, 100, 110],
- 'max_leaves': [10, 20, 30, 40, 50]
- ‘alpha’: [0.01, 0.1, 1.0, 10.0, 100.0]
- ‘lambda’: [0.01, 0.1, 1.0, 10.0, 100.0]

**
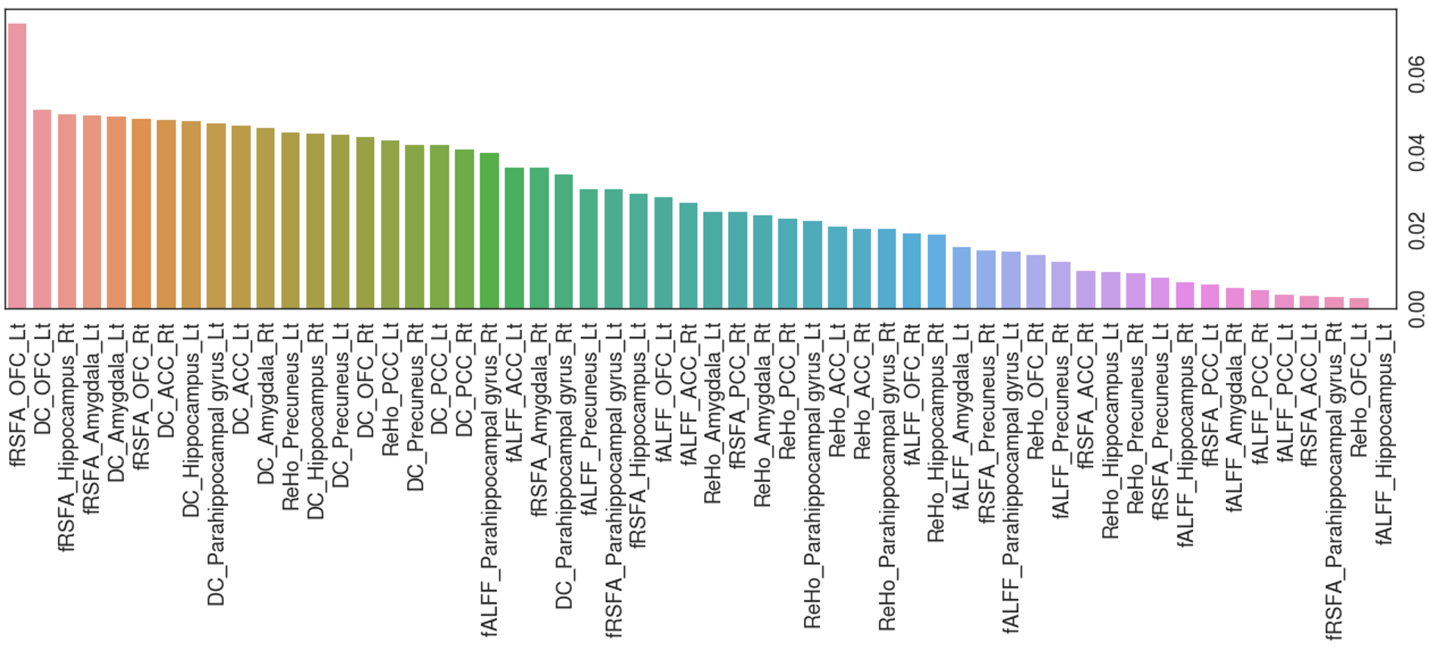
Comparison of SHAP Feature Importance with Coefficient Weights**

**Figure S1.** Coefficient weights bar plot of all input resting-state brain functional radiomic features. The values represent the weights of the eXtreme Gradient Boosting (XGBoost) model. Abbreviations: Rt, right; Lt, left; DC, degree centrality; fRSFA, fractional resting-state physiological fluctuation amplitude; fALFF, fractional amplitude of low-frequency fluctuation; ReHo, regional homogeneity; PCC, posterior cingulate cortex; OFC, orbitofrontal cortex; ACC, anterior cingulate cortex.

As a complementary analysis of the feature importance of the trained XGBoost model, we evaluate the coefficient weights of the linear estimators from the XGBoost model which can also reflect the level of feature importance. It can be seen from Figure S1 that the coefficient weight across the functional radiomic features share a highly similar pattern to that of the SHAP feature importance (Figure 4). Pearson’s correlation analysis between the two values resulted in a significant correlation (r = 0.981, *p* < 0.001) which suggests robustness of the feature importance with respect to the different methodology (Figure S2).

**
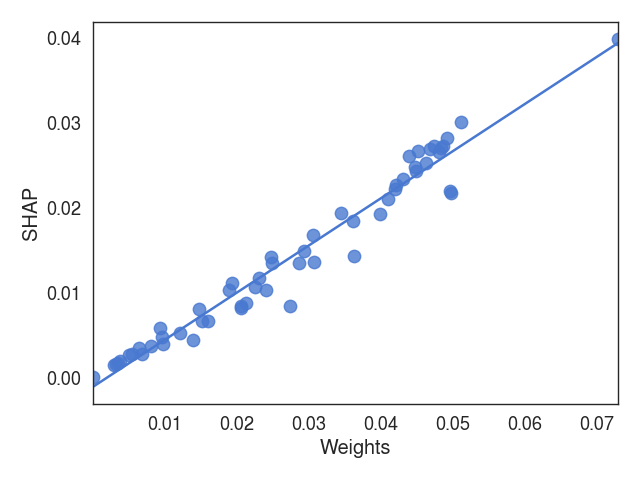
Figure S2.** Scatter plot of the coefficient weights and the SHAP values of the trained XGBoost model. The regression line is fitted with the least-squares estimation.

**References**

1. Abraham, A.; Pedregosa, F.; Eickenberg, M.; Gervais, P.; Mueller, A.; Kossaifi, J.; Gramfort, A.; Thirion, B.; and Varoquaux, G. Machine Learning for Neuroimaging with Scikit-Learn. Frontiers in Neuroinformatics 2014, 8, 14.
2. Avants, B.B.; Epstein, C.L.; Grossman, M.; Gee, J.C. Symmetric diffeomorphic image registration with cross-correlation: Evaluating automated labeling of elderly and neurodegenerative brain. Medical Image Analysis 2008, 12, 26–41.
3. Yashar, B.; Restom, K.; Liau, J.; Liu, T.T. A component based noise correction method (CompCor) for BOLD and perfusion based fMRI. Neuroimage 2007, 37, 90–101.
4. Dale, A.M.; Fischl, B.; Sereno, M.I. Cortical surface-based analysis: I. Segmentation and surface reconstruction. Neuroimage 1999, 9, 179–194.
5. Oscar, E.; Blair, R.; Markiewicz, C.J.; Berleant, S.L.; Moodie, C.; Ma, F.; Isik, A.I.; et al. “FMRIPrep.” Software. Zenodo 2018.https://doi.org/10.5281/zenodo.852659.
6. Oscar, E.; Blair, R.; Markiewicz, C.J.; Blair, R.W.; Moodie, C.; Isik, A.I.; Aliaga, A.E.; Kent, J.; Goncalves, M.; DuPre, E.; Snyder, M.; Oya, H.; Ghosh, S.S.; Wright, J.; Durnez, J.; Poldrack, R.A.; Gorgolewski, K.J. fMRIPrep: A robust preprocessing pipeline for functional MRI. Nature Methods 2019, 16, 111-116.
7. Fonov, V.S.; Evans, A.C.; McKinstry, R.C.; Almli, C.R.; and Collins, D.L. Unbiased nonlinear average age-appropriate brain templates from birth to adulthood. Neuroimage 2009, 47 supplement 1, S102.
8. Gorgolewski, K., Burns, C.D.; Madison, C.; Clark, D.; Halchenko, Y.O.; Waskom, M.L.; Ghosh, S. Nipype: A flexible, lightweight and extensible neuroimaging data processing framework in Python. Frontiers in Neuroinformatics 2011, 5, 13.
9. Gorgolewski, K.J., Esteban, O.; Markiewicz, C.J.; Ziegler, E.; Ellis, D.G.; Notter, M.P.; Jarecka, D.; et al. Nipype. Software. Zenodo 2018. <https://doi.org/10.5281/zenodo.596855>.
10. Greve, D.N; Fischl, B. Accurate and robust brain image alignment using boundary-based registration. Neuroimage 2009, 48, 63–72.
11. Jenkinson, M; Bannister, P; Brady, M; Smith, S. Improved optimization for the robust and accurate linear registration and motion correction of brain images. Neuroimage 2002, 17, 825–841.
12. Klein, A.; Ghosh, S.S.; Bao, F.S.; Giard, J.; Häme, Y.; Stavsky, E.; Lee, N.; Rossa, B.; Reuter, M.; Chaibub Neto, E.; Keshavan, A. Mindboggling morphometry of human brains. PLOS Computational Biology 2017, 13, e1005350.
13. Lanczos, C. Evaluation of noisy data. Journal of the Society for Industrial and Applied Mathematics Series B Numerical Analysis 1964, 1, 76–85.
14. Power, J.D.; Mitra, A.; Laumann, T.O.; Snyder, A.Z.; Schlaggar, B.L.; Petersen, S.E. Methods to detect, characterize, and remove motion artifact in resting state fMRI. Neuroimage 2014, 84, 320–341.
15. Satterthwaite, T.D.; Elliott, M.A.; Gerraty, R.T.; Ruparel, K.; Loughead, J.; Calkins, M.E.; Eickhoff, S.B.; Hakonarson, H.; Gur, R.C.; Gur, R.E.; Wolf, D.H. An improved framework for confound regression and filtering for control of motion artifact in the preprocessing of resting-state functional connectivity data. Neuroimage 2013,64, 240-256.
16. Tustison, N.J.; Avants, B.B.; Cook, P.A.; Zheng, Y.; Egan, A.; Yushkevich, P.A.; Gee, J.C. N4ITK: Improved N3 bias correction. IEEE Transactions on Medical Imaging 2010, 29, 1310–1320.
17. Zhang, Y.; Brady, M.; Smith, S. Segmentation of brain MR images through a hidden Markov random field model and the expectation-maximization algorithm.” IEEE Transactions on Medical Imaging 2001, 20, 45–57.
18. Zang, Y.; Jiang, T.; Lu, Y.; He, Y.; Tian, L. Regional homogeneity approach to fMRI data analysis. Neuroimage 2004, 22(1), 394-400.
19. Zou, Q.H.; Zhu, C.Z.; Yang, Y.; Zuo, X.N.; Long, X.Y.; Cao, Q.J.; ... Zang, Y.F. An improved approach to detection of amplitude of low-frequency fluctuation (ALFF) for resting-state fMRI: fractional ALFF. Journal of Neuroscience Methods 2008, 172(1), 137-141.
20. Kannurpatti, S.S.; Biswal, B.B. Detection and scaling of task-induced fMRI-BOLD response using resting state fluctuations. Neuroimage 2008, 40(4), 1567-1574.
21. Kannurpatti, S.S.; Biswal, B.B. Prediction of task-related BOLD fMRI with amplitude signatures of resting-state fMRI. Frontiers in Systems Neuroscience 2012, 6, 7.
22. Craddock, R.C.; Clark, D.J. Optimized implementations of voxel-wise degree centrality and local functional connectivity density mapping in AFNI. BioRxiv 2016, 067702.
